# Neuropathologic findings and age‐related differences in Finnish pediatric medico‐legal autopsies

**DOI:** 10.1111/1556-4029.70307

**Published:** 2026-03-08

**Authors:** Elias Hakanen, Petteri Oura, Paula Katriina Vauhkonen

**Affiliations:** ^1^ Department of Forensic Medicine University of Helsinki Helsinki Finland; ^2^ Forensic Medicine Unit Finnish Institute for Health and Welfare Helsinki Finland

**Keywords:** autopsy, forensic neuropathology, forensic pathology, pediatric autopsy, SIDS, SUDC, sudden infant death syndrome, sudden unexpected death in childhood, sudden unexpected death in infancy, SUDI, SUID

## Abstract

Neuropathological examination plays a critical role in medico‐legal cause‐of‐death investigation, especially in determining the cause and manner of death in pediatric autopsies. Although a comprehensive neuropathological examination is recommended, limited data exists of the diagnostic yield of neuropathology consultations in such cases. This retrospective register‐based study reviewed Finnish pediatric medico‐legal autopsies from 2016 to 2022 involving full neuropathological examination performed by a neuropathologist, with a focus on age‐related differences in consultation rates, themes, and diagnostic findings. Data were retrieved from the medico‐legal cause‐of‐death investigation documents, and included background characteristics, neuropathologic diagnoses, and causes and manners of death. A total of 118 pediatric medico‐legal autopsies with a neuropathology consultation were performed during the study period. The consultation rate was highest among infants (aged <1 year; 45.6%), compared to early childhood (aged 1–10 years; 23.0%) and late childhood (aged 11–18 years; 6.2%). Traumatic brain injury was the most frequent consultation theme overall (33.9%), while the frequency of epilepsy‐related consultations increased with age. Hypoxic‐ischaemic neuronal injury constituted the commonest finding, with an overall prevalence of 43.2%. Findings associated with childhood epilepsy were more prevalent in early and late childhood, with epilepsy accounting for 15.2% of deaths in the latter group. Positive diagnostic findings were uncommon among infants, consistent with a high proportion of cases (42.1%) classified as sudden infant death syndrome. These findings provide forensic pathologists with age‐specific diagnostic insights, supporting more targeted resource use and neuropathology consultation practices.

**Highlights**

- Neuropathology consultation rate was highest among infants and declined thereafter.
- Traumatic brain injury was the most common consultation theme.
- Neuropathological diagnoses varied across pediatric age groups.
- Positive diagnostic findings were uncommon among infants.
- Acknowledging the age‐relatedness of neuropathologic diagnoses may improve consultation protocols.

## 1 INTRODUCTION

Evaluation of the central nervous system (CNS) plays a significant role in forensic medicine. Neuropathological examination aids in distinguishing between traumatic and non‐traumatic CNS injuries, as well as in identifying underlying medical conditions predisposing to death [1]. The developing CNS in children (i.e. aged 0–18 years [2]) is especially susceptible to a wide range of pathological conditions. The exclusion of such conditions is essential in cases of sudden unexpected death in childhood (SUDC, aged 12 months and older), sudden unexpected death in infancy (SUDI; also termed *sudden unexpected infant death [SUID]*), below the age of 12 months, encompassing sudden infant death syndrome (SIDS) [3], and especially in cases of suspected abusive head trauma [4].

The distribution of causes and manners of death varies significantly with age. Globally, leading causes of childhood mortality include pneumonia, diarrhea, and malaria [5], whereas in countries with low child mortality rates, congenital abnormalities and complications of preterm birth are more prominent [6]. In forensic settings, both natural causes (e.g. infections), as well as unnatural causes, and SIDS are found to explain infant demise [7]. SIDS typically peaks between 2 and 4 months of age [8], while unexplained SUDC is most commonly declared in children aged 1–4 years [9]. In later childhood, unnatural causes of death become increasingly prevalent [5, 10]. These aforementioned differences are reflected in the neuropathologic findings, which tend to vary depending on the age of the decedent [11].

Previous research highlights the importance of neuropathology specialist consultation not only in the diagnosis of traumatic brain injury (TBI), but also in detecting congenital and developmental disorders [12]. In all cases of SUDI and SUDC, diligent evaluation of the CNS is recommended [13, 14, 15], and neuropathology referral is encouraged to reduce the risk of incomplete diagnostic assessment and misinterpretation of the pathological findings [15]. However, neuropathology consultation availability and practices may vary across countries and institutions. In Finland, the decision to request a neuropathology consultation in pediatric deaths is made by the forensic pathologist on a case‐by‐case basis. To date, the diagnostic yield of these consultations and their value to the cause‐of‐death investigation have not been evaluated within Finnish forensic practice.

The aim of this retrospective register‐based study was to review neuropathologic findings of pediatric medico‐legal autopsies conducted in Finland over the period 2016–2022, in which full neuropathological examination was performed by a neuropathologist. Potential differences in neuropathology consultation rates, themes, and diagnoses were explored across different pediatric age groups, and the significance of neuropathology consultation was assessed in light of the most commonly observed diagnoses in infancy and later childhood.

## 2 MATERIALS AND METHODS

### 2.1 Protocol

In Finland, a medico‐legal investigation into the cause(s) and manner of death is required when there is no recent medical history to account for the death; when the death is sudden and unexpected; and when there is suspicion of a non‐natural cause of death (i.e., accident, suicide, homicide, occupational disease, complication of medical or surgical care, or war) [16]. The authority to initiate such an investigation and request a medico‐legal autopsy lies with the police.

Requested in approximately 15% of all deaths in Finland, medico‐legal autopsies are carried out by forensic pathologists at one of the five regional offices of the Forensic Medicine Unit, under the jurisdiction of the Finnish Institute for Health and Welfare [17]. Each office is responsible for autopsies within its designated area: Southern Finland, Southwestern Finland, Western and Inland Finland, Eastern Finland, and Northern Finland including Lapland. A centralized electronic information system has been used since 2016 to handle and record case files, including consultation referrals and statements from ancillary examinations, such as neuropathology.

This study was retrospective and register‐based, building on a nationwide sample of medico‐legal autopsies conducted between 2016 and 2022 for children and adolescents aged 18 years and younger, in which a full neuropathological examination was performed by a neuropathologist. Data were retrieved from the electronic information system and included background characteristics, neuropathologic findings, and causes and manners of death. For comparison, the total number of medico‐legal autopsies conducted for individuals aged ≤18 years during the same period was also collected. The study received approval from the Finnish Institute for Health and Welfare (THL/1802/6.02.00/2023).

### 2.2 Characteristics of the sample

The age (in years) and sex (male or female) of each decedent were obtained from the autopsy order submitted by the police. To ensure meaningful age categories, the decedents were classified into three groups: infants (<1 year), early childhood (1–10 years), and late childhood (11–18 years). These definitions were based on guidelines established by the World Health Organization and the United Nations, with correspondence to age groups suggested by Krous [2, 3, 18].

The reason(s) for requesting a medico‐legal autopsy were extracted from the police's written autopsy request. The indications were as follows: no previous medical history; sudden and unexpected death; suspicion of homicide, accident, suicide, or medical or surgical adverse event. Several could be selected for a single case.

Measurements of body height (cm), weight (kg), and fresh brain weight (g) were collected from the medico‐legal autopsy report. The brain weight included the cerebrum, cerebellum, brainstem, and leptomeninges. The postmortem interval (PMI; in days) was calculated by subtracting the estimated date of death from the date of autopsy.

The consultation theme(s) were retrieved from the neuropathology referral, which was formulated by the forensic pathologist. The themes were classified as follows: TBI, epilepsy, hypoxia‐ischaemia, and other/not specified. Several could be selected for a single case.

Causes and manners of death were recorded from the official death certificate, issued by the forensic pathologist at the conclusion of the investigation. Natural disease‐related deaths were classified as having a manner of death of “disease”, while deaths resulting from external or unknown causes were assigned a manner of death from the following options: accident, homicide, suicide, complication of medical or surgical care, or undetermined. SIDS and unexplained SUDC were declared according to a broad international consensus, i.e., thorough death scene investigation by the police authority, review of clinical history, and complete medico‐legal autopsy with ancillary examinations (including radiological imaging and histological, toxicological, biochemical, and microbiological investigations) failing to establish a sufficient cause of death. The extent of ancillary investigations in each case varied to a small degree, according to the examining forensic pathologist. By definition, all SIDS and unexplained SUDC cases were categorized under the undetermined manner of death [19].

Underlying causes of death were categorized according to the 10th revision of the International Statistical Classification of Diseases and Related Health Problems (ICD‐10), such that each category contained a minimum of five cases. The following categories were formed: asphyxia (T17, T71, T75.1); injury (S00‐T14, T20‐T35, T90‐T95); poisoning (T36‐T65); infectious disease (A00‐B99, J00‐J22, U07); perinatal condition (P00‐P96); epilepsy (G40‐G41); other neurological disorder (G04, G10‐G37, G43‐G99); endocrine and metabolic disease (E00‐E89); congenital malformation (Q00‐Q99); SIDS (R95); and other. Finally, the proportion of cases, in which an acute head injury (S00‐S09, T04.0, T06.0) was identified as either the underlying or a contributing cause of death, was calculated.

### 2.3 Neuropathologic diagnoses

Brains were fixed in formaldehyde and delivered to the local neuropathology department for examination. The extent and approach of the examination, including both gross inspection and histological sampling, were performed at the discretion of the neuropathologist.

For this study, neuropathologic diagnoses were retrieved from the diagnosis line of the consultation report. A list of the individual diagnoses, as well as categories of the most frequent diagnoses, is presented in the results section. The absence of any findings that would justify a neuropathologic diagnosis was also documented. Diagnoses were retrieved as listed in the diagnosis line of the neuropathologist's report; there was no minimum threshold for the severity of the findings, provided that the finding appeared in the diagnosis line. Mere observations mentioned only in the narrative portion of the report were excluded. In the case of hemorrhages, it was common practice for the consulting neuropathologists to report only the type (e.g., subdural or intracerebral) but not commit to an etiological diagnosis (e.g., traumatic or non‐traumatic).

### 2.4 Statistical analysis

Statistical analyses were conducted using SPSS Statistics version 29 (IBM, Armonk, NY, USA). Descriptive statistics included percentages and frequencies for categorical variables and medians with interquartile ranges (IQRs) for continuous variables. To demonstrate differences in background characteristics (sex, PMI, neuropathology consultation themes) and neuropathological diagnoses between the three age groups, the Fisher–Freeman–Halton exact test was used for categorical variables, and the Mann–Whitney U test was applied to continuous variables. A p‐value of 0.05 was considered the threshold for statistical significance.

## 3 RESULTS

A total of 118 pediatric medico‐legal autopsies underwent neuropathology consultation, which corresponds to 15.2% of all medico‐legal autopsies of decedents aged ≤18 years over the study period. Consultation rate was markedly higher among infants (57/125, 45.6%), in comparison to the older age groups (early childhood 28/122, 23.0%, late childhood 33/529, 6.2%), with some annual and regional variation (Table S1).

Table 1 presents the background characteristics of the sample. Male sex was overrepresented in all age groups (63.6% of cases). The most common indication for medico‐legal autopsy across all age groups was sudden and unexpected death (65.3%). The median PMI was 4 days and the shortest among infants (3 days, IQR 2–6). The most prevalent consultation theme across all age groups was TBI (infants 38.6%, early childhood 25.0%, late childhood 33.3%), followed by hypoxia‐ischaemia (19.3%, 17.9%, 15.2%, respectively). The frequency of epilepsy‐related neuropathology consultation was lower among infants than in later childhood (infants 1.8%, early childhood 25.0%, late childhood 21.2%). In the rest of the sample (48.3%), consultation themes were miscellaneous or non‐specified.

**TABLE 1:** Background characteristics of the sample.

Characteristic	ALL (*n* = 118)	Age group (years)			*p* value
		< 1 (*n* = 57)	1–10 (*n* = 28)	11–18 (*n* = 33)	
Sex					
Male	63.6 (75)	63.2 (36)	64.3 (18)	63.6 (21)	
Female	36.4 (43)	36.8 (21)	35.7 (10)	36.4 (12)	1.000
Indication for medico‐legal autopsy (multiple options)					
No previous medical history	58.5 (69)	61.4 (35)	46.4 (13)	63.6 (21)	
Sudden and unexpected death	65.3 (77)	78.9 (45)	50.0 (14)	54.5 (18)	
Suspicion of…					
Homicide	12.7 (15)	10.5 (6)	14.3 (4)	15.2 (5)	
Accident	11.0 (13)	3.5 (2)	17.9 (5)	18.2 (6)	
Suicide	4.2 (5)	0.0 (0)	0.0 (0)	15.2 (5)	
Medical or surgical adverse event	11.9 (14)	10.5 (6)	17.9 (5)	9.1 (3)	
Postmortem interval (days)^a^	4 (3–7)	3 (2–6)	6 (3–7)	6 (4–9)	0.002
Autopsy measurements					
Body height (cm)^a^	87 (55–147)	55 (50–62)	104 (94–119)	167 (151–176)	
Body weight (kg)^a^	11.5 (4.4–31.0)	4.4 (3.2–6.1)	18.0 (14.3–21.0)	59.0 (44.5–79.0)	
Brain weight (g)^a^	1157 (595–1443)	577 (395–688)	1329 (1200–1440)	1544 (1277–1626)	
Neuropathology consultation theme (multiple options)					
Traumatic brain injury	33.9 (40)	38.6 (22)	25.0 (7)	33.3 (11)	0.484
Epilepsy	12.7 (15)	1.8 (1)	25.0 (7)	21.2 (7)	<0.001
Hypoxia‐ischaemia	17.8 (21)	19.3 (11)	17.9 (5)	15.2 (5)	0.951
Other/not specified	48.3 (57)	47.4 (27)	50.0 (14)	48.5 (16)	1.000

*Note*: Numbers are frequencies (*n*) with prevalences (%).

^a^ Median (interquartile range).

Table 2 provides a breakdown of the neuropathologic findings within the sample. Comparisons between the age groups in terms of the most frequent diagnoses are shown in Table 3. Across all age groups, hypoxic‐ischaemic neuronal injury constituted the most prevalent diagnosis (infants 31.6%, early childhood 64.3%, late childhood 45.5%). The prevalence of oedema exhibited a small age‐related increase (infants 19.3%, early childhood 25.0%, late childhood 36.4%), and evidence of brain herniation was observed primarily in late childhood (infants 0%, early childhood 3.6%, late childhood 21.2%) (Table 3).

**TABLE 2:** Breakdown of neuropathologic diagnoses among the sample.

Diagnosis	ALL (*n* = 118)	Age group (years)		
		<1 (*n* = 57)	1–10 (*n* = 28)	11–18 (*n* = 33)
Hypoxic‐ischaemic neuronal injury	51	18	18	15
Oedema	30	11	7	12
Herniation	8		1	7
Hydrocephalus	1		1	
Atrophy				
Cerebral	1			1
Hippocampus	4		3	1
Malformation				
Chiari 1	1			1
Holoprosencephaly	1		1	
Lissencephaly	1		1	
Macrencephaly	6	2	3	1
Polymicrogyria	3	1	1	1
Focal cortical dysplasia	2		1	1
Hippocampal malformation	1		1	
Pontine hypoplasia	1		1	
Aqueductal stenosis	1			1
Cerebral anomaly (CATCH 22)	1			1
Venous angioma	1		1	
Neoplasm/non‐neoplastic lesion				
Arachnoid cyst	2		2	
Cerebral cyst	1			1
Pineal cyst	1			1
Spinal teratoma	1			1
Infarct				
Acute	2		2	
Old	2			2
Leukoencephalopathy	3	1	1	1
Hepatic encephalopathy	1			1
Encephalitis	2	1		1
Lysosomal storage disorder	2			2
Cerebral microcalcification	1	1		
Acute hemorrhage				
Subdural	3	2		1
Subarachnoid	9	2	2	5
Intracerebral	8	1	3	4
Intraventricular	4	2	1	1
Other/unspecified	1	1		
Traumatic brain injury				
Contusion	2			2
Traumatic axonal injury	6	1	1	4
Hypoglycaemic brain injury	2			2
No diagnostic changes	26	25		1

*Note*: Numbers are frequencies (*n*). Comparisons between the age groups in terms of the most frequent diagnoses are shown in Table 3. One case may have several diagnoses.

**TABLE 3:** Comparisons between the age groups in terms of the most frequent neuropathologic diagnoses.

Diagnosis	ALL (*n* = 118)	Age group (years)			*p* value
		< 1 (*n* = 57)	1–10 (*n* = 28)	11–18 (*n* = 33)	
Hypoxic‐ischaemic neuronal injury	43.2 (51)	31.6 (18)	64.3 (18)	45.5 (15)	0.016
Edema	25.4 (30)	19.3 (11)	25.0 (7)	36.4 (12)	0.198
Herniation	6.8 (8)	0.0 (0)	3.6 (1)	21.2 (7)	<0.001
Malformation	14.4 (17)	5.3 (3)	28.6 (8)	18.2 (6)	0.010
Neoplasm/non‐neoplastic lesion	4.2 (5)	0.0 (0)	7.1 (2)	9.1 (3)	0.041
Any acute hemorrhage	11.9 (14)	7.0 (4)	14.3 (4)	18.2 (6)	0.261
Traumatic axonal injury	5.1 (6)	1.8 (1)	3.6 (1)	12.1 (4)	0.095
No diagnostic changes	22.0 (26)	43.9 (25)	0.0 (0)	3.0 (1)	<0.001

*Note*: Numbers are frequencies (*n*) with prevalences (%). One case may have several diagnoses.

The third‐largest diagnostic group was malformations, diagnosed seldom in infants (5.3%) or in late childhood (18.2%) but in nearly one third of early childhood autopsies (28.6%). The most prevalent malformation in the sample was macrencephaly (6 cases), followed by polymicrogyria (3 cases) (Table 2). In addition, individual cases of various other malformations were observed.

The fourth most prevalent diagnostic group was acute hemorrhages, observed across all age groups (infants 7.0%, early childhood 14.3%, late childhood 18.2%), with subarachnoid hemorrhage being the most common type. Other, less common diagnostic groups included traumatic axonal injury (TAI) (6 cases) and neoplastic/non‐neoplastic lesions (5 cases) (Table 3).

Notably, in nearly half of the infants (43.9%), no diagnostic neuropathologic changes were identified. In contrast, all early childhood autopsies and nearly all (97.0%) late childhood autopsies yielded some neuropathologic diagnosis. Furthermore, the neuropathologic findings among infants were primarily non‐specific, such as hypoxic‐ischaemic neuronal injury and oedema. In addition, only a few other neuropathologic diagnoses were observed (macrencephaly *n* = 2, polymicrogyria *n* = 1, leukoencephalopathy *n* = 1, encephalitis *n* = 1, cerebral microcalcification *n* = 1, acute hemorrhages *n* = 8, TAI *n* = 1) (Table 2).

As shown in Table 4, diseases accounted for the majority of deaths in the sample (46.6%). In nearly half of the infants (42.1%), the underlying cause of death was SIDS, raising the prevalence of undetermined manner of death in this age group (50.9%). Common natural causes of death among infants were perinatal conditions (17.5%), followed by infectious diseases (12.3%). In early childhood, natural causes of death were more divergent, with neurological disorders (other than epilepsy) constituting the commonest cause (14.3% of cases). In late childhood, the most common natural cause of death was epilepsy (15.2%), followed by other neurological disorders and endocrine/metabolic disease (9.1% in each).

**TABLE 4:** Causes and manners of death among the sample.

	ALL (*n* = 118)	Age group (years)		
		<1 (*n* = 57)	1–10 (*n* = 28)	11–18 (*n* = 33)
Manner of death				
Disease	46.6 (55)	40.4 (23)	60.7 (17)	45.5 (15)
Accident	13.6 (16)	3.5 (2)	21.4 (6)	24.2 (8)
Homicide	7.6 (9)	3.5 (2)	7.1 (2)	15.2 (5)
Suicide	3.4 (4)	0.0 (0)	0.0 (0)	12.1 (4)
Undetermined	25.4 (30)	50.9 (29)	0.0 (0)	3.0 (1)
Complication of medical or surgical care	3.4 (4)	1.8 (1)	10.7 (3)	0.0 (0)
Underlying cause of death				
Unnatural				
Asphyxia	11.0 (13)	5.3 (3)	21.4 (6)	12.1 (4)
Injury	10.2 (12)	3.5 (2)	7.1 (2)	24.2 (8)
Poisoning	5.1 (6)	0.0 (0)	0.0 (0)	18.2 (6)
Natural				
Infectious disease	9.3 (11)	12.3 (7)	10.7 (3)	3.0 (1)
Perinatal condition	8.5 (10)	17.5 (10)	0.0 (0)	0.0 (0)
Epilepsy	5.9 (7)	0.0 (0)	7.1 (2)	15.2 (5)
Other neurological disorder	6.8 (8)	1.8 (1)	14.3 (4)	9.1 (3)
Endocrine and metabolic disease	4.2 (5)	0.0 (0)	7.1 (2)	9.1 (3)
Congenital malformation	5.1 (6)	3.5 (2)	10.7 (3)	3.0 (1)
Sudden infant death syndrome	20.3 (24)	42.1 (24)	0.0 (0)	0.0 (0)
Other^a^	13.6 (16)	14.0 (8)	21.4 (6)	6.1 (2)
Acute head injury as a cause of death				
Underlying	5.9 (7)	3.5 (2)	3.6 (1)	12.1 (4)
Contributory	0.8 (1)	0.0 (0)	0.0 (0)	3.0 (1)

*Note*: Numbers are frequencies (*n*) with prevalences (%).

^a^ All remaining causes of death, regardless of the manner of death.

The proportion of deaths due to violent manners increased with age. Among infants, accidents and homicides accounted for 7.0% of all deaths. In early childhood, the proportion rose to 28.6% and further increased to 39.4% in late childhood. Suicides were reported only in late childhood, comprising 12.1% of deaths in this age group. Asphyxia, injuries, and poisoning constituted 26.3% of the deaths in the sample. Acute head injury was fairly uncommon as an underlying (5.9%) or contributory (0.8%) cause of death. Notably, there were no unexplained deaths in the older age groups, as there was only one undetermined manner of death in an adolescent with either accidental or intentional poisoning.

## 4 DISCUSSION

In this study, we conducted a retrospective review of 118 pediatric medico‐legal autopsy cases that underwent a full neuropathological examination by a neuropathologist, with a primary focus on identifying age‐related differences in consultation rates, themes, and diagnostic findings. We found differences in consultation rates, which were highest among infants (45.6%), followed by early childhood autopsies (23%). In contrast, neuropathology consultation was sought in only 6.2% of late childhood autopsies. These differences likely reflect variations in forensic practices related to the age of the deceased. In infants, consultation is often a standard procedure due to a cautious approach and possibly limited training in neuropathology for the independent assessment of immature brains. In contrast, in late childhood, the CNS is more developed and externally resembles that of an adult, allowing forensic pathologists to perform examinations with greater confidence, thereby reducing the frequency of neuropathology consultations.

Natural causes (mainly infections and perinatal conditions) accounted for 40.4% of infant deaths, with SIDS contributing an additional 42.1%. The reported rates of SIDS in medico‐legal autopsies varies across countries and time periods, principally due to divergence in reporting and coding these deaths [19]. In a Canadian regional study, SIDS was attributed as the cause of death in 66% of medico‐legal infant autopsies conducted between 1995 and 2005 [20]. In contrast, a retrospective 30‐year population‐based study from the Basque Country (1991–2020) also reported SIDS in 66% of all SUDIs, but this number included cases that had not undergone a medico‐legal autopsy [21]. There has also been a terminological shift from SIDS toward the broader category of SUDI/SUID, reflecting increased recognition of diagnostic heterogeneity in these deaths [19]. In early childhood, natural causes explained 60.7% of deaths, and in late childhood, 45.5%, with epilepsy being the predominant cause. These results closely align with previous forensic literature [22]. Age‐related increase in violent manners of death was also observed, consistent with previous research [9]. It is estimated that approximately 20% of SUDCs are eventually explained after a comprehensive autopsy, with causes commonly including complications of acute infections, such as myocarditis, pneumonia, or encephalitis, as well as conditions like congenital anomalies, diabetes, and cancer [9]. In our sample, there were no unexplained causes of death in early or late childhood. This is likely due to the rather small sample size, as unexplained SUDC is a rare entity [9, 22].

Non‐specific neuropathologic findings, most notably hypoxic‐ischemic neuronal injury and oedema, were the most frequently observed diagnoses across the entire sample, consistent with prior studies conducted in adult populations [12, 23]. Although common in both natural and unnatural deaths, their secondary nature limits their diagnostic value. The third‐largest diagnostic group was malformations, diagnosed in nearly one‐third of early childhood autopsies, followed by acute hemorrhages, with the highest prevalence observed in late childhood. Although TBI constituted the commonest consultation theme across all age groups, it was neuropathologically confirmed in only 6.8% of cases, and notably, only in one infant. On the other hand, the data did not allow for a clear distinction between traumatic and non‐traumatic hemorrhages, meaning that the proportion of intracranial injuries in the dataset may be higher than the number of TBIs suggests.

The frequency of both epilepsy‐related consultations and epilepsy deaths increased with age, accounting for 15.2% of deaths in late childhood. This aligns with previous research indicating that early‐onset epilepsy is linked to an elevated risk of sudden unexpected death in epilepsy (SUDEP), particularly in individuals experiencing frequent generalized tonic–clonic seizures [24]. The risk of SUDEP in children is up to ten times lower than in adults, ranging from 1.1 to 3.4 per 10,000 patient‐years [24]. Childhood epilepsy arises from a diverse range of etiologies, including perinatal insults, neoplasms, TBI, infections, cortical malformations, and genetic predispositions. Common neuropathologic findings associated with childhood epilepsy include focal cortical dysplasia and hippocampal sclerosis [11, 24, 25, 26]. In our data, few such abnormalities were diagnosed in total. While the precise pathophysiology of SUDEP remains unclear, several mechanisms have been proposed, primarily implicating cardiac, respiratory, and autonomic dysfunction, either independently or in combination [27]. Emerging evidence also suggests potential shared pathophysiology between SUDEP, SIDS, and SUDC, including hippocampal disorganization, particularly altered thickness and irregular configuration of the dentate gyrus [24]. In our sample, only one case of hippocampal malformation was identified, which is significantly fewer than previously reported. For instance, a study by McGuone et al. reported hippocampal abnormalities in 95% of SUDC autopsies, mainly in the dentate gyrus [11], while Kinney et al. found these in 70% of autopsied SUDI and SUDC cases [28]. One possible explanation here could be differences in reporting neuropathologic findings on a diagnostic level. It should also be noted that such findings in neuropathological examination do not independently suffice to determine the cause of death, and these are not specific to SIDS or unexplained SUDCs [11, 29].

Although infants exhibited the highest consultation rate, nearly half of the cases revealed no diagnostic neuropathologic changes. This result suggests that positive diagnostic findings are uncommon in this age group and aligns with 42.1% of cases in our sample classified as SIDS – a diagnosis made by exclusion. In a retrospective review of 885 SUDI‐related postmortem examinations, published by Pryce et al., only 6% of deaths were eventually attributed to neuropathologic causes—most identifiable through clinical history or gross examination [30]. In fewer than 1% of cases, histological neuropathology alone established the cause of death in the absence of neurological history or clear macroscopic abnormalities [30]. However, no standardized method currently exists that reliably maximizes diagnostic yield while minimizing the risk of misinterpretation [15]. Moreover, establishing a diagnosis of exclusion requires not only thorough investigation but also sufficient expertise to reliably determine the absence of findings [19].

In our sample of infants, the majority of the neuropathologic findings have been detectable either during gross examination (hemorrhages, macrencephaly, polymicrogyria) or through comprehensive inspection of sectioned tissue, particularly when conducted on fixed brain [1, 31]. In contrast, the diagnosis of TAI requires histopathological analysis using β‐amyloid precursor protein immunostaining, but this procedure may be carried out independently by the forensic pathologist [1, 32]. Leukoencephalopathies and encephalitides are significant findings in the determination of cause of death, and their diagnosis also relies on histological examination. However, these conditions are frequently preceded by neurological symptoms, which may prompt the forensic pathologist to suspect an underlying neurological disorder and to consult a neuropathologist at a lower threshold. Our findings thus seem to support previous literature, suggesting it may be appropriate to limit infant neuropathology consultations to cases where the brain exhibits macroscopic abnormalities or where the clinical history strongly suggests a neurological cause of death, and other investigations fail to reveal a definitive explanation for demise [30, 33]. However, independent evaluation of the immature CNS requires adequate training and expertise in neuropathology among forensic pathologists. In Finland, all suspected SIDS/SUDC cases are primarily investigated by a forensic pathologist – who is also solely responsible for determining the cause and manner of death in these cases. Instead of full neuropathology outsourcing, focused training to strengthen neuropathological competence could represent a cost‐effective strategy for limiting external referrals and associated diagnostic delays [34].

This study has several limitations that should be acknowledged. First, not all pediatric cases were systematically referred for neuropathology consultation, and the consultation rates were particularly low in the oldest age group. This weakens the generalizability of the observed neuropathologic findings to late childhood medico‐legal autopsies. Second, the examinations were carried out by various specialists across different regions of Finland. There may be subjective variation in determining which diagnoses are considered important or certain enough to be included in the diagnosis line – especially in cases with a prolonged PMI, introducing variability in the diagnoses. Last, as the sample presents medico‐legal cause‐of‐death investigations, these findings may not be extrapolated to medical autopsies or used for estimating prevalences of causes or manners of death in the general Finnish pediatric population.

## 5 CONCLUSION

This study reported 118 neuropathologically examined pediatric autopsy cases of the Finnish medico‐legal authority over the period 2016–2022. Our primary objective was to explore age‐related differences in consultation rates, themes, and diagnostic findings and to assess the added value of neuropathology consultations across age groups. The consultation rate was highest among infants. While TBI was the most common consultation theme, the frequency of epilepsy‐related consultations increased with age. Hypoxic‐ischaemic neuronal injury constituted the commonest finding. Positive diagnostic findings were uncommon among infants. Findings associated with childhood epilepsy were notably more common in later childhood. These findings provide forensic pathologists with valuable insights into age‐specific diagnostic expectations. Recognizing age‐related differences in current consultation practices and diagnostic findings may inform decisions regarding resource allocation, prioritize neuropathological training, and support further development of autopsy and consultation protocols. Future research should aim to expand the dataset by increasing the representation of older pediatric age groups, thereby enabling a more comprehensive understanding of neuropathologic findings across these populations.

## FUNDING INFORMATION

This work was part of the Finnish Translational Forensic Neuropathology Project (FINNE), funded by the Jane and Aatos Erkko Foundation.

## CONFLICT OF INTEREST STATEMENT

The authors have no conflicts of interest to delcare.

## Supporting information


Table S1.

## Data Availability

The data that support the findings of this study are available from Finnish Institute for Health and Welfare. Restrictions apply to the availability of these data, which were used under license for this study. Data are available from the author(s) with the permission of Finnish Institute for Health and Welfare.

## References

[1] Kalimo H , Saukko P , Graham D . Neuropathological examination in forensic context. Forensic Sci Int. 2004;146(2–3):73–81. 10.1016/j.forsciint.2004.06.022 15542266

[2] United Nations . Convention on the rights of the child. Adopted by the general assembly, 20 November 1989. United Nations Treaty Series 1577:3. New York, NY: United Nations; 1989.

[3] Krous HF . Sudden infant death syndrome (SIDS), sudden unexpected death in infancy (SUDI), and sudden unexplained death in childhood (SUDC). In: Byard R , Collins K , editors. Forensic pathology of infancy and childhood. New York, NY: Springer; 2014. p. 193–206.

[4] Narang SK , Haney S , Duhaime A‐C , Martin J , Binenbaum G , Campomanes AG d A , et al. Abusive head trauma in infants and children: technical report. Pediatrics. 2025;155(3):e2024070457. 10.1542/peds.2024-070457 39992695

[5] Roth G , Abate D , Abate K . Global, regional, and national age‐sex‐specific mortality for 282 causes of death in 195 countries and territories, 1980–2017: a systematic analysis for the global burden of disease study 2017. Lancet. 2018;392(10159):1736–1788. 10.1016/S0140-6736(18)32203-7 30496103 PMC6227606

[6] Liu L , Oza S , Hogan D , Chu Y , Perin J , Zhu J , et al. Global, regional, and national causes of under‐5 mortality in 2000–15: an updated systematic analysis with implications for the sustainable development goals. Lancet. 2016;388(10063):3027–3035. 10.1016/S0140-6736(16)31593-8 27839855 PMC5161777

[7] Weber MA , Ashworth MT , Risdon RA , Hartley JC , Malone M , Sebire NJ . The role of post‐mortem investigations in determining the cause of sudden unexpected death in infancy. Arch Dis Child. 2008;93(12):1048–1053. 10.1136/adc.2007.136739 18591183

[8] Kaler J , Hussain A , Lee S . Manifestation and pathogenesis of sudden infant death syndrome: a review. Crit Rev Eukaryot Gene Expr. 2020;30(2):111–120. 10.1615/CritRevEukaryotGeneExpr.2020033009 32558490

[9] Wojcik MH , Krous HF , Goldstein RD . Sudden unexplained death in childhood: current understanding. Pediatr Emerg Care. 2023;39(12):984–985. 10.1097/01.pec.0000997588.40847.b0 38019719 PMC10688964

[10] Feld K , Feld D , Quandel K , Banaschak S . Manner of death, causes of death and autopsies in infants, children and adolescents. Rechtsmedizin. 2022;32(6):465–472. 10.1007/s00194-022-00568-y

[11] McGuone D , Leitner D , William C , Faustin A , Leelatian N , Reichard R , et al. Neuropathologic changes in sudden unexplained death in childhood. J Neuropathol Exp Neurol. 2020;79(3):336–346. 10.1093/jnen/nlz136 31995186 PMC7036658

[12] Oura P , Viitasalo V , Mäkinen H , Hakkarainen AJ . Breakdown and significance of neuropathology consultations in medico‐legal autopsies in southern Finland between 2016 and 2022. Forensic Sci Int. 2025;370:112466. 10.1016/j.forsciint.2025.112466 40209631

[13] Pinneri K , Matshes EW . Recommendations for the autopsy of an infant who has died suddenly and unexpectedly. Acad Forensic Pathol. 2017;7(2):171–181. 10.23907/2017.019 31239972 PMC6474526

[14] Bajanowski T , Vege Å , Byard RW , Krous HF , Arnestad M , Bachs L , et al. Sudden infant death syndrome (SIDS)—standardised investigations and classification: recommendations. Forensic Sci Int. 2007;165(2–3):129–143. 10.1016/j.forsciint.2006.05.028 16806765

[15] Bundock EA , Bruce DA , Castellani RJ , Devinsky O . Evaluation for central nervous system disorders. In: Bundock E , Corey T , Andrew T , editors. Unexplained pediatric deaths: investigation, certification, and family needs. San Diego, CA: Academic Forensic Pathology International; 2019. 35107904

[16] Kuvaja P , Pakanen L , Alajärvi S . How the cause of death is established in Finland. Suom Laakaril. 2018;73:1749–1751.

[17] Oura P , Eklin A , Sajantila A . Neuropathology consultation rates in medico‐legal autopsies show substantial within‐country variation—a nationwide Finnish study. Forensic Sci Res. 2023;8(3):198–201. 10.1093/fsr/owad027 38221966 PMC10785601

[18] Dattani S . How do statistical organizations define age periods for children? Our World in Data; 2023. Available from: https://ourworldindata.org/how‐do‐statistical‐organizations‐define‐age‐periods‐in‐children. Accessed 25 Jul 2025.

[19] Goldstein RD , Blair PS , Sens MA , Shapiro‐Mendoza CK , Krous HF , Rognum TO , et al. Inconsistent classification of unexplained sudden deaths in infants and children hinders surveillance, prevention and research: recommendations from the 3rd international congress on sudden infant and child death. Forensic Sci Med Pathol. 2019;15(4):622–628. 10.1007/s12024-019-00156-9 31502215 PMC6872710

[20] Hanna S , Rao C , Fernandes JR . A retrospective study of sudden infant death syndrome (SIDS) cases in the Hamilton‐Wentworth region from 1995 to 2005. J Can Soc Forensic Sci. 2007;40(2):39–52. 10.1080/00085030.2007.10757150

[21] Belmonte E , Monzó A , Morentin B . Epidemiological changes and causes of sudden unexpected death in infancy: a 30‐year population‐based study in Basque Country. Rev Esp Med Legal. 2023;49(1):11–19. 10.1016/j.reml.2022.06.002

[22] Burton EC , Singer NA . Pediatric natural deaths. In: Byard R , Collins K , editors. Forensic pathology of infancy and childhood. New York, NY: Springer; 2014. p. 855–898.

[23] Barranco R , Bonsignore A , Ventura F . Immunohistochemistry in postmortem diagnosis of acute cerebral hypoxia and ischemia. Medicine (Baltimore). 2021;100(25):e26486. 10.1097/MD.0000000000026486 34160462 PMC8238305

[24] Morse AM , Kothare SV . Pediatric sudden unexpected death in epilepsy. Pediatr Neurol. 2016;57:7–16. 10.1016/j.pediatrneurol.2016.01.004 26850125

[25] Dahl‐Hansen E , Koht J , Syvertsen M . Epilepsy at different ages—etiologies in a Norwegian population. Epilepsia Open. 2019;4(1):176–181. 10.1002/epi4.12292 30868128 PMC6398108

[26] Jordan RD , Coscia M , Lantz P , Harrison W . Sudden unexpected death in epilepsy. Am J Forensic Med Pathol. 2022;43(3):259–262. 10.1097/PAF.0000000000000773 35642769

[27] Tolstykh GP , Cavazos JE . Potential mechanisms of sudden unexpected death in epilepsy. Epilepsy Behav. 2013;26(3):410–414. 10.1016/j.yebeh.2012.09.017 23305781

[28] Kinney HC , Poduri AH , Cryan JB , Haynes RL , Teot L , Sleeper LA , et al. Hippocampal formation maldevelopment and sudden unexpected death across the pediatric age spectrum. J Neuropathol Exp Neurol. 2016;75(10):981–997. 10.1093/jnen/nlw075 27612489 PMC6281079

[29] McGuone D , Crandall LG , Devinsky O . Sudden unexplained death in childhood: a neuropathology review. Front Neurol. 2020;11:582051. 10.3389/fneur.2020.582051 33178125 PMC7596260

[30] Pryce JW , Paine SML , Weber MA , Harding B , Jacques TS , Sebire NJ . Role of routine neuropathological examination for determining cause of death in sudden unexpected deaths in infancy (SUDI). J Clin Pathol. 2012;65(3):257–261. 10.1136/jclinpath-2011-200264 22135027

[31] Katelaris A , Kencian J , Duflou J , Hilton JM . Brains at necropsy: to fix or not to fix? J Clin Pathol. 1994;47(8):718–720. 10.1136/jcp.47.8.718 7962624 PMC502144

[32] Dolinak D , Reichard R . An overview of inflicted head injury in infants and young children, with a review of β‐amyloid precursor protein immunohistochemistry. Arch Pathol Lab Med. 2006;130(5):712–717. 10.5858/2006-130-712-AOOIHI 16683890

[33] Hutchins KD , Menke J , Reichard RR . The utility of routine brain histology in forensic cases. Acad Forensic Pathol. 2012;2(1):42–45. 10.23907/2012.006

[34] Oura P , Mäkinen H , Ruotsalainen R , Ruokomäki M , Virtanen A , Hakkarainen AJ . First year of in‐house forensic neuropathology consultations in Helsinki, Finland. Int J Legal Med. 2025;139(2):805–815. 10.1007/s00414-024-03399-6 39804481 PMC11850508

