# Supplementary material for: Neuropathologic findings and age‐related differences in Finnish pediatric medico‐legal autopsies
Source: J Forensic Sci. 2026 Mar 8;71(3):1246–54. doi: 10.1111/1556-4029.70307 (PMC13139811; doi:10.1111/1556-4029.70307)
Supplement: Supplementary file 1 — Table S1. [file JFO-71-1246-s001.docx]

TABLE S1 Annual and regional distributions of cases included in the present sample (i.e., medico-legal autopsies of decedents aged ≤ 18 years associated with a neuropathology consultation). Corresponding annual and regional distributions of all medico-legal autopsies of decedents aged ≤ 18 years are also included.

|  | All medico-legal autopsies of decedents aged ≤ 18 years | | |  | Age group (years) | | | | | | | | | | |
| --- | --- | --- | --- | --- | --- | --- | --- | --- | --- | --- | --- | --- | --- | --- | --- |
|  |  |  |  |  | < 1 | | |  | 1-10 | | |  | 11-18 | | |
|  | N_1_ | N_2_ | % |  | N_1_ | N_2_ | % |  | N_1_ | N_2_ | % |  | N_1_ | N_2_ | % |
| Year |  |  |  |  |  |  |  |  |  |  |  |  |  |  |  |
| 2016 | 13 | 97 | 13.4 |  | 7 | 15 | 46.7 |  | 5 | 22 | 22.7 |  | 1 | 60 | 1.7 |
| 2017 | 23 | 119 | 19.3 |  | 8 | 20 | 40.0 |  | 6 | 19 | 31.6 |  | 9 | 80 | 11.3 |
| 2018 | 15 | 110 | 13.6 |  | 9 | 20 | 45.0 |  | 1 | 14 | 7.1 |  | 5 | 76 | 6.6 |
| 2019 | 26 | 128 | 20.3 |  | 13 | 27 | 48.1 |  | 6 | 19 | 31.6 |  | 7 | 82 | 8.5 |
| 2020 | 16 | 126 | 12.7 |  | 6 | 12 | 50.0 |  | 4 | 14 | 28.6 |  | 6 | 100 | 6.0 |
| 2021 | 16 | 102 | 15.7 |  | 12 | 19 | 63.2 |  | 2 | 14 | 14.3 |  | 2 | 69 | 2.9 |
| 2022 | 9 | 94 | 9.6 |  | 2 | 12 | 16.7 |  | 4 | 20 | 20.0 |  | 3 | 62 | 4.8 |
| Region |  |  |  |  |  |  |  |  |  |  |  |  |  |  |  |
| Southern Finland | 51 | 309 | 16.5 |  | 27 | 46 | 58.7 |  | 11 | 52 | 21.2 |  | 13 | 211 | 6.2 |
| Southwestern Finland and Åland | 28 | 86 | 32.6 |  | 14 | 15 | 93.3 |  | 5 | 8 | 62.5 |  | 9 | 63 | 14.3 |
| Western and Inland Finland | 21 | 161 | 13.0 |  | 6 | 25 | 24.0 |  | 6 | 20 | 30.0 |  | 9 | 116 | 7.8 |
| Eastern Finland | 8 | 89 | 9.0 |  | 4 | 20 | 20.0 |  | 3 | 15 | 20.0 |  | 1 | 54 | 1.9 |
| Northern Finland and Lapland | 10 | 131 | 7.6 |  | 6 | 19 | 31.6 |  | 3 | 27 | 11.1 |  | 1 | 85 | 1.2 |
| All years and regions | 118 | 776 | 15.2 |  | 57 | 125 | 45.6 |  | 28 | 122 | 23.0 |  | 33 | 529 | 6.2 |

N_1_ = Number of medico-legal autopsies associated with a neuropathology consultation (i.e., the present sample)
N_2_ = Number of all medico-legal autopsies performed during the corresponding year/in the corresponding region
% = Percentage of autopsies associated with a neuropathology consultation (N_1_) relative to all medico-legal autopsies (N_2_) during the corresponding year/in the corresponding region
